# The Direct and Indirect Relationships of Environmental, Interpersonal and Personal Factors with High School Students Physical Activity: An Ecological Approach

**DOI:** 10.3390/ijerph18030874

**Published:** 2021-01-20

**Authors:** Brigita Mieziene, Arunas Emeljanovas, Ilona Tilindiene, Laura Tumynaite, Laima Trinkuniene, Ichiro Kawachi

**Affiliations:** 1 Department of Physical and Social Education, Lithuanian Sports University, 44221 Kaunas, Lithuania; arunas.emeljanovas@lsu.lt (A.E.); ilona.tilindiene@lsu.lt (I.T.); laura.tumynaite@stud.lsu.lt (L.T.); laima.trinkuniene@lsu.lt (L.T.); 2 Department of Social and Behavioral Sciences, Harvard T.H. Chan School of Public Health, Harvard University, Boston, MA 02115, USA; ikawachi@hsph.harvard.edu

**Keywords:** moderate-to-vigorous physical activity, social capital, physical activity motivation, neighborhood safety

## Abstract

Background: Across countries, young people are not sufficiently physically active. The evidence confirms that beyond demographic and individual agents, individuals participate within their social and physical environment. The ecological model enables a search for the modifiable factors in specific populations, as it allows consideration of factors affecting individuals’ lives on different levels, as well as considering the interplay of those factors. The aim of this study was to examine the complex interconnections among environmental, social capital and motivational factors at different levels, within an ecological model for high school students’ moderate-to-vigorous physical activity during their leisure time. Methods: This cross-sectional population-based study included 1285 students from 14 to 18 years old, with a mean age of 16.14 ± 1.22. Physical activity, neighborhood physical activity recourses, neighborhood safety, social capital, physical activity motivation and sociodemographic factors were evaluated. Logistic regression, mediation and moderation analyses were performed predicting moderate-to-vigorous physical activity during leisure time. Results: In the final multivariate logistic regression model, greater social participation (OR 1.03 [1.01–1.05]), higher relative autonomy index (OR 1.11 [1.06–1.15]) and male gender (OR 1.71 [1.13–2.57]) directly predicted meeting MVPA recommendations. Any significant moderation effects (*p* > 0.05) of environmental characteristics were not found for the relationship between social capital, motivational factors and moderate-to-vigorous physical activity. The evidence of positive indirect mediation effects was found in all five models for social capital components as all CIs for its βs do not contain 0, though standardized effect sizes were between 0.02 and 0.07, indicating small effect sizes. Conclusion: These findings provide support for the presence of some direct and indirect pathways from social capital to moderate-to-vigorous physical activity. Future intervention strategies should focus on strengthening physical activity motivation by encouraging the development of social network and social participation as well as family, neighborhood and school social capital within the framework of the ecological model.

## 1. Introduction

Extensive evidence shows that a sufficient level of physical activity (PA) lowers the risk for premature all-cause mortality and the incidence of many chronic diseases by 20–30%, a higher amount and greater intensities are related to greater health benefits [1]. Higher versus lower PA was more favorably associated not only with physical, but also psychological, social and cognitive health indicators in children and youth [2]. Meanwhile, across the countries, there is evidence that young people are not sufficiently physically active, that is, only around 20% of school aged children from 37 countries meet requirements of sufficient physical activity [3]. Researchers propose that the decline in PA starts at age 7 [4], and by the age of 16 to 18 it becomes very low [5]. Moreover, results of the recent meta-analysis indicate that over the transition from adolescence to adulthood moderate-to-vigorous physical activity (MVPA) declines by approximately 13–17% of the baseline value, measured both subjectively and objectively [6]. So, adolescence is a vulnerable period for establishing and maintaining sufficient PA for further life. To achieve this, many researches are searching for factors related to sufficient PA.

Research found that PA in adolescents varies depending on a number of factors starting from the most studied demographics. For instance, male gender, higher parental education and socioeconomic status relate to higher chances of sufficient physical activity [7]. Further, among PA determinants, personal motivation to be physically active was also confirmed in many studies as the most proximal predictor of PA [8]. However, motivation also constantly decreases with age along with PA [9]. Beyond these individual-level indicators, interpersonal factors like social networks, social participation and social support, playing the role of reinforcing factors, increase the probability of higher physical activity [10]. On the other hand, there is a proposition that PA decline might be associated with social transitions in adolescents’ lives [6]. However, the social environment not only provides emotional, financial, instrumental and other kinds of support, but PA itself could be a part of socialization as family, friends’ ties could be strengthened by common physical activities. Although, studies demonstrated that the impact of interpersonal factors for PA are mediated by other factors at the individual level [11]. So, physical activities provide the right set of circumstances to strengthen social ties [12] in all main social contexts, which in adolescence could be defined as family, neighborhood and school. Alongside this, research also paid attention to the enabling role of PA friendly physical environment, which has a moderation effect for PA through interpersonal factors. For example, neighborhood safety is related to higher PA for those adolescents who spend less time with friends, and, by contrast, neighborhood safety for those who spend more time with friends was associated with greater engagement in sedentary behavior [13]. A systematic literature review showed that the infrastructure for walking, cycling in the neighborhood could induce demand for walking and cycling [14]. 

Summing up the writing above, this evidence confirms that beyond demographic and individual agents, individuals are active participants within their social and physical environment [15]. However, authors argue that studies aiming to identify determinants of health behaviors have almost exclusively been limited to examination of those separate elements as mentioned above. Studies showing the “bigger picture” are needed, which capture the track of relationships within and between the different type of possible behavioral predictors [16].

Explaining adolescents’ PA, there is a well-known but not much used ecological model, which focuses on the interaction between, and interdependence of, factors within and across levels in the model like intrapersonal, interpersonal, environmental, policy levels [17]. Authors argue that factors within and across levels of an ecological model may differently affect behavior depending on the age of population and the behavior itself [18]. The ecological model also enables a search for the modifiable factors in specific populations. Theoretical premises of the ecological model state the following. First, predisposing, personal factors, such as attitudes, motivation towards PA, make an impact on the PA participation related decision-making processes. Second, the reinforcing interpersonal factors encourage participation through the social environment and include significant others such as parents, teachers and peers. Third, enabling environmental factors such as access to PA resources are supposed as necessary but not sufficient determinants of physical activity. Finally, demographic factors (such as age, gender) directly affect how an individual will assimilate various influences. 

One study explored the interactive effects of the school physical environment and school social capital on the MVPA of students while at school. They found that both school social capital and school physical environment were associated with MVPA, however school social capital may be a more important factor in increasing students MVPA than the school physical environment [19]. Meanwhile, the interaction of physical, social environment and individual motivation predicting PA outside of school was not examined in any other study.

The aim of this study was to examine the complex interconnections among environmental, social capital and motivational factors laying on different levels within an ecological model for high school students’ leisure time MVPA. Specifically, the focus was controlling for sociodemographic factors (1) to determine environmental, interpersonal, personal factors associated with high school students’ meeting recommendations for moderate-to-vigorous physical activity during leisure time; and (2) to examine whether neighborhood physical activity resources and neighborhood safety moderates the associations between motivation for physical activity, social capital components and students’ moderate-to-vigorous physical activity during leisure time; (3) to identify the mediation effect of motivation for physical activity in the indirect relationship between social capital components and moderate-to-vigorous physical activity during leisure time.

## 2. Materials and Methods 

### 2.1. Study Design and Procedure

This is cross-sectional population-based study. The study sample was selected across all 10 regions of Lithuania within the period of September–November 2019 and represents both urban (61.1%) and rural (38.9%) areas. Cluster (area) random sampling was used. Within each of the ten regions two randomly selected schools (primary sampling units) were selected: one from the main city in the region and one from the rural area. Two schools which refused to participate in the study were replaced with similar schools in the same region. Twenty schools were selected in total. Then, within each selected school, students from the 9th, 10th, 11th and 12th grade, one class per grade, were chosen using the classes whose number was followed by the letter A (e.g., 10A). Finally, all students in the selected schools and grades were included in the study. Both the school and classes in the schools were considered clusters. Selected students were given a questionnaire to fill. The filling questionnaire took approximately 30 min.

### 2.2. Participants

Initially, the study included 1386 students. Sixty-five questionnaires (4.7%) were not returned, and 36 questionnaires (2.6%) were inconsistently or inaccurately filled or deliberately damaged and were not suitable for analysis. Finally, data of 1285 students were included for analysis. The students’ age varied from 14 to 18 years old, with a mean and standard deviation (SD) 16.14 ± 1.22, respectively. Among them 42.2 were male (Table 1). Those whose parents gave their written consent and those students who gave their verbal consent were given the study questionnaires. The study was conducted in accordance with the Declaration of Helsinki, and the protocol was approved by the Ethics Committee of Lithuanian Sports University (No. SMTEK-13). Students anonymously completed the paper questionnaires in the classroom. Researchers explained the aim and procedures of the study before the questionnaires were completed.

### 2.3. Measurements

#### 2.3.1. Moderate-to-Vigorous Physical Activity

The assessment of MVPA was based on its definition by the World Health Organization (WHO) which states that moderate physical activity noticeably accelerates and vigorous physical activity substantially increases the heart rate (WHO) [20]. The examples of moderate activities (like brisk walking or bicycling) and vigorous activities (such as jogging, aerobic dancing and bicycling uphill) were provided along with the two open items that were given to identify MVPA. The students were asked to identify (1) how often, by identifying the exact numbers of times/week, and (2) how long, by identifying hours and/or minutes per bout, they participated in the listed physical activities or activities of similar intensity within the last 7 days during their leisure time, out of school. Then, the number of minutes spent in MVPA was totaled and converted to hours per week. The continuous MVPA variable was used in moderation analysis to determine interaction effects. Responses on MVPA were also dichotomized into ≥7 h per week and ˂7 h/week, according to the physical activity guidelines for school age children (WHO) and represented those who meet PA recommendations and those who do not, respectively. The dichotomized MVPA variable was used for multivariate logistic regression analysis to predict meeting MVPA recommendations.

#### 2.3.2. Environmental Factors

Following other studies [21] to evaluate Neighborhood PA resources students were asked if there are playgrounds, parks or gyms close to their home or if they can easily access them. Neighborhood safety was assessed with a single item by asking if it is difficult to walk or jog in the neighborhood because of things like traffic, no sidewalks, dogs or gangs. Answers for both items were dichotomized for 1—“Yes”, and 2—“No”. Then the answer for neighborhood safety was reversed in order that an answer coded by 1 would indicate a positive response.

#### 2.3.3. Interpersonal Factors

The assessment of social capital in adolescents in this study was based on theoretical premises [22,23] and previous empirical research [24,25,26]. Cognitive social capital in the contexts of family, neighborhood and school was evaluated by 14 items with the answers on a Likert scale, with 1 meaning “totally disagree” and 5 meaning “totally agree”. The six items in family context evaluated the family support, perceived enhancement of sense of autonomy and self-determination by parents, e.g., “Do you feel that your family understands and cares about you”? In a context of neighborhood social support, social trust and informal social control was evaluated by three items, e.g., “Do you feel people trust each other in your neighborhood”? School social capital was assessed by 5 items in terms of vertical trust, horizontal trust, reciprocity, communication, e.g., “Do you think students collaborate with each other in your high school”?

An exploratory structural equation modeling (ESEM) was used to determine structure of the instrument. In a factorial analysis of 14 statements, three subscales emerged: family social capital (6 statements; Cronbach α 0.905), neighborhood social capital (2 statements; Cronbach α 0.757) and school social capital (5 statements; scale compatibility factor Cronbach α 0.853). One statement which was supposed to belong to neighborhood social capital subscale (“Do people in the neighborhood tolerate inappropriate behavior by your peers?”) was removed after factor analysis, because it did not correlate at least ≥0.40 with any of the factors. ESEM indicators confirmed the factor structure (chi-square (χ2) = 268.66; degrees of freedom (df) = 41; root mean square error of approximation (RMSEA) = 0.069; the comparative fit index (CFI) = 0.974; Tucker–Lewis index (TLI) = 0.950; the standardized root mean square residual (SRMR) = 0.022). Items on all subscales were combined into a mean score. 

Social network, which is a measure of a number of friendship connections was evaluated by one item “Please rate how many people you are interacting with, could you name as your friends?” and social participation, which represented the total number of contacts a person has with other individuals over a certain period of time, with a single item “How many times in the last month, have you been doing anything with your friends (e.g., going to the movies, taking a walk, otherwise entertaining)”?

The median of each of the five social capital variables was used as a cut off to binarize them into lower and higher representation of the corresponding variable in order to use these dichotomized variables in the moderation analysis.

#### 2.3.4. Personal Factors 

To assess the underlying students’ motivation to participate in exercise and physical activities the BREQ-2 questionnaire was used [27]. The 19-item questionnaire is comprised of five subscales that reflect intrinsic, identified, introjected, external behavior regulation and amotivation. The questionnaire was double-translated, reviewed, piloted and approved by the experts in the field. Answers were provided on a 5-point Likert scale ranging from 0 = “not true for me” to 4 = “very true for me” used to rate each item. Subscales were calculated by summing scores on their belonging items and averaging by the number of items. In this study we used The Relative Autonomy Index (RAI) score which indicates the degree to which a student’s physical activity motivation is autonomous. The RAI is calculated by multiplying each subscale score by a specific ratio, and then summing the rated scores. The RAI ranges from −24 to +20, with higher positive scores indicating more autonomous motivations [28]. In this study of Lithuanian adolescents, the confirmatory factor analysis (CFA) was performed and demonstrated good parameters (χ2 = 262.64; df = 86; RMSE = 0.042 [0.036–0.048]; CFI = 0.993; TLI = 0.986; SRMR = 0.014) confirming the structure of the original scales. The Cronbach α of each scale ranged from 0.817 to 0.899. The median of the RAI variable was used as a cut off to binarize it into “Lower” and “Higher” motivation. This dichotomized variable was used for the moderation analysis. 

#### 2.3.5. Socio-Demographic Factors 

Parental education was indicated by the parents choosing among the answers “Elementary school”, “Middle school”, “High school”, “Vocational training”, “College” and “University”. Then, answers were dichotomized, by 1 indicating lower than college education and 2 indicating college or university education. Answers for both parents of each student were provided along with the written consent for their children to participate in the study, unless the student lived with a single parent. 

Place of living was evaluated by students indicating if they live in an urban or a rural area. 

Gender was evaluated by providing two options for answering: 1—male; and 2—female. 

Age was indicated by asking students to answer to open question “How old are you”? The answers were provided in full years at the time of survey.

As there was not the focus in this study to determine the effect of gender and age as it was determined in many previous studies, gender and age were allocated among the socio-demographic factors instead for personal and were considered as covariates. 

### 2.4. Statistical Analysis

Data were analyzed using SPSS 24.0 (SPSS Inc., Chicago, IL, USA) and MPLUS 8.4 [29] software. Exploratory Structural Equation Modeling (ESEM) was performed to explore structure on social capital instrument. Confirmatory factor analysis was used to confirm the structure of the Breq-2 questionnaire in the Lithuanian sample of adolescents. All continuous variables used in analyses met the assumptions of normality of the distribution after outliers exceeding three SDs were removed and scales’ skewness and kurtosis were within the range—1 and 1. Descriptive statistics were calculated to determine the means and SDs and frequencies of variables used in the study. The relationships between moderate to vigorous physical activity and environmental, interpersonal, personal and sociodemographic factors were identified using univariate and multivariate logistic regression analysis, producing odds ratios (ORs) and 95% confidence intervals (95% CIs). The associations between the dependent and each independent cluster of variables were entered separately into the model (four models for environmental, interpersonal, personal and sociodemographic factors). The associations between dependent and all independent variables were calculated by entering clusters of variables simultaneously into the model, the Enter method was used. The univariate general linear model was used for moderation analysis. For mediation analysis, PROCESS version 3.5. [30] SPSS macro (Model 4) was employed, which tests direct and indirect effects in mediation model. Confidence intervals (CI) of 95% were estimated. An effect was considered significant when the CI did not include zero. Bootstrapping was set at 5000 samples. The completely standardized indirect effects were calculated as effect sizes for mediation [31]. Their values of 0.01, 0.09 and 0.25 representing small, medium and large effect sizes, respectively [32]. Statistical significance was set at a ***p***-value of less than 0.05.

STROBE Statement—checklist guidelines were followed in organizing this paper.

## 3. Results

### 3.1. Prediction of Meeting Moderate to Vigorous Physical Activity Recommendations from Factors within the Ecological Model

Descriptive statistics in Table 1 revealed that only 16.3% of adolescents meet the recommendation to be physically active at a moderate to vigorous level for at least 60 min/day. The vast majority (approximately four out of five adolescents) of high school students perceive their neighborhood environment as physical activity-friendly in terms of accessibility to PA resources and three out of four declared that it is safe enough in the neighborhood to be physically active. Descriptive analysis of interpersonal characteristics revealed that adolescents in their social network on average have six friends and count, on average, social participation in activities of any kind nine times per month. Scores on family, neighborhood and school social capital were all above the possible average in the range of 1 to 5. Autonomous motivation for physical activity (RAI) ranged within −15 and 20 points with 7.28 points on average. Students in a sample were within 14 and 18 years old with a mean of around 16 years. There were more girls than boys (42.2 versus 57.8%). Roughly two thirds of them lived in an urban area, the rest 38.9% lived in a rural area. About two thirds of their mothers and almost a half of their fathers had a college or university degree (Table 1). 

Univariate logistic analysis constructed to predict meeting the PA recommendation (≥7 h/week) of MVPA was performed per block of environmental, interpersonal, personal and sociodemographic variables. Results of this analysis showed that recommended MVPA is directly predicted by high versus low neighborhood PA resources, high versus low neighborhood safety, a greater social network and more occasions of social participation, a higher score on the family social capital scale, higher RAI and male gender. In a multivariate logistic regression (Table 1) blocks of predictors were entered simultaneously in the Table 1 listed order starting from the most distal within the ecological model—environmental factors—to the personal factors. In the final model sociodemographic factors were included as the covariates. So, in the final model greater social participation, higher relative autonomy index and male gender directly predicted recommended MVPA of ≥7 h/week (Table 1). In the multivariate model, when personal factor—RAI—was included, previously significant predictors of PA environmental factors, social network and family social capital stopped being significant.

### 3.2. Examination of Moderation Effect 

**Figure 1:**
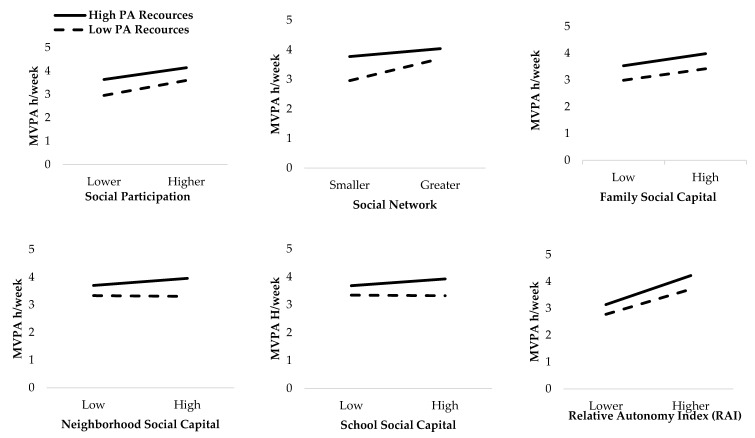
Checking for moderating effect of physical activity (PA) resource availability on the relationship between social capital and motivation characteristics and hours of weekly adolescent MVPA. All interactions are non-significant (*p* > 0.05).

**Figure 2:**
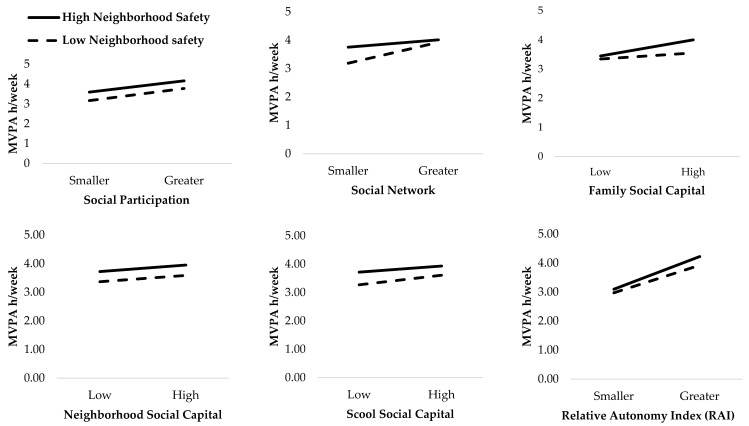
Checking for moderating effect of neighborhood safety on the relationship between social capital and motivation characteristics and hours of weekly adolescent MVPA. All interactions are non-significant (*p* > 0.05).

Further, interaction effects between each environmental characteristic and each social capital and motivation characteristics to predict MVPA were added into the separate univariate general linear models, which were adjusted by gender. However, no significant moderation effects (*p* > 0.05) of environmental characteristics for the relationship between social capital, motivation factors and MVPA were found (Figure 1 and Figure 2).

### 3.3. Examination of Mediation Effect

Finally, analysis examining RAI mediation effect in the relationship between social capital components and MVPA as a continuous variable was performed (Table 2). All mediation models for each social capital variable satisfied the criteria for mediation that the predictor variable should be significantly related to the mediator and the mediator should be significantly related to the outcome variable. All social capital components showed significant positive associations with mediator RAI (Table 2). As in the previous logistic regression analysis, predicting a binarized indicator of meeting MVPA recommendations, this analysis also replicated direct relationships of social participation and social network with continuous MVPA; greater social network and social participation were related to more accumulated hours of MVPA. Significant in predicting binarized MVPA, family social capital was not directly related to continuous MVPA. However, the evidence of positive indirect effect was found in all five models for social capital components, as all CIs for its βs do not contain 0. RAI significantly mediated the relationship between MVPA and social network; social participation; social capital in family, neighborhood, school contexts, though standardized effect sizes were between 0.02 and 0.07, indicating small effect sizes. Nevertheless, these findings provide support for the presence of some direct and indirect pathways from social capital to MVPA.

## 4. Discussion

The study aimed to examine direct and indirect correlates of high school students’ leisure time MVPA within the ecological model. Toward this end, we sought to identify how perceived PA resources and neighborhood safety on an environmental level, social capital components on interpersonal level and PA motivation on a personal level were related with high school students’ MVPA, controlling for the main sociodemographic factors. 

The univariate analysis revealed that environmental factors—higher perceived PA recourses and greater neighborhood safety—were related to meeting MVPA recommendations. Another study supports our findings showing that the more PA recourses there are at adolescents’ disposal the higher is their physical activity [33]. However, the latter study found no associations of PA with neighborhood safety [33]. Another study found that school physical environment was positively associated with student MVPA at school [19]. The previous review of studies on the topic showed inconsistent results, in some cases depending on the type of the measurement. When both neighborhood environmental attributes and physical activity were measured objectively, less significant associations in the expected direction between them were found than when the measurements were self-reported. Specifically, results of the review supported association between pedestrian safety, structures (e.g., traffic lights, crosswalks) and reported physical activity. However, there were some inconsistencies in relationships of PA with access to parks, recreation facilities and street connectivity [34]. Anyway, the relationships between leisure MVPA and neighborhood environment in the current study lost their significance in the multivariate analysis when a personal factor, specifically PA motivation, was included. That led to the premise that an interplay might be possible between environmental and personal factors predicting physical activity. A multilevel study in 29 European countries found that, besides other environmental factors like lower annual average national temperatures, lower average national income and weaker physical education policies, adolescents do more MVPA in countries with higher perceptions of community safety. The vigorous PA is higher where there is a higher percentage of urban areas among other factors [35]. 

Among interpersonal–social capital components, greater social network, social participation and higher family social capital were significantly associated with meeting MVPA recommendations in a univariate analysis independently of other social capital factors. The social network is considered as a source, which provides coping resources that can promote physical activity participation in the form of suggestions to join peers to go to sports club or certain training sessions, or assistance with starting a physical activity program [36]. The importance of the family social capital could be explained assuming that family members promote physical activity in their children through the mechanisms of modelling, joint participation and support [37,38], which might be emotional, instrumental (by driving to training sessions) or financial (by paying for training sessions). Although adolescence is the period when peers account for more and more importance, it seems that family social capital still has its impact for health-related behavior [39,40]. The results of the current study only partly overlap with results in the study of the Croatian adolescents, but only in the context of family, where social support was a significant predictor of PA. They also found that social capital within neighborhood and school contexts appeared to be important for PA among youth [40]. The relationship was found among school social capital and students’ MVPA at school in a sample of Canadian school students as well [19], which was not observed in the current study. Further in the current study, in the multivariate analysis, only social participation of all social capital components emerged as an independent predictor. The effect of social network in terms of a number of friendship connections for MVPA might be affected by the norms regarding the physical activity behaviors of those friends’ groups. So, in the multivariate analysis, its effect was transferred by PA motivation which is generally increased when supported by peers [41]. However, social participation, in terms of engagement in various activities together with friends, most probably includes physical activities among other participation, so the latter still directly predicts MVPA even controlling for PA motivation. That’s why social participation remained significant after adjusting analysis for other factors. Previous research review concludes that friendship plays an important role in shaping physical activity behaviors. Peer groups might have utility as a means of increasing youth physical activity in the interventions [42]. In the current study, neither neighborhood nor school social capital was related to MVPA. Although, it is commonly supposed and confirmed by many studies that, in particular, the school social capital, in terms of social support or support for autonomy at school, plays an important role shaping school students’ leisure physical activity [8]. In the current study, social capital measurement was not specifically aimed to measure PA-related support or encouragement, instead it covered a general understanding of trust, collaboration and reciprocity among students and teachers and between the students themselves at school. Similarly generalized was social capital in other contexts; this is probably why there were no direct relationships, as that kind of social interaction strengthens students’ personal factors such as self-efficacy or motivation for many kinds of behaviors [43]. Meanwhile, family social capital was not related, and only school social capital was important for Turkish students’ PA, surprisingly inversely—the higher the teacher–students interpersonal trust the lower the students’ PA [44]. 

Further, the examination of moderation effects of environmental factors for the relationship between MVPA and interpersonal as well as personal factors revealed that any of environmental factors significantly affected relationships among interpersonal, personal factors and PA in the sample of adolescents. Similar results were found in Canada among more than 18,000 6–10th grade students. That study did not indicate an interaction effect between the school physical environment and school social capital for MVPA at school [19]. Although, Rhodes et al. [45] hypothesized that individuals with lower levels of PA motivation may benefit from the presence of a PA-friendly physical environment, because they then have fewer barriers to overcome in order to engage in PA, results of this study did not confirm that. Neither high PA recourses nor perceived neighborhood safety interplayed with PA motivation to increase MVPA. However, other empirical studies and theoretical considerations are in line with Rhodes’ and his colleagues’ hypothesis [33,46]. The lack of studies failing to confirm the interplay between PA supportive physical environment and personal factors or social environment within the ecological framework might perhaps also be attributed to publication bias, as nonsignificant findings might not have been reported [16].

Finally, PA motivation as a personal factor, was a significant predictor of meeting MVPA recommendations in the univariate analysis, and remained significant in the multivariate analysis independently of environmental and interpersonal factors. The important role of PA motivation for PA is confirmed in many studies among children and adolescents [47,48]. PA motivation was also an important predictor for PA for healthcare students [49]. As PA motivation is the most proximal PA predictor as it lays on the personal level within the framework of the ecological model, its mediation effect was examined in the relationship between social capital components and MVPA. In the multivariate analysis when PA motivation was added, among interpersonal social capital factors only social participation remained significant. This again suggests that PA motivation as a personal and most proximal to behavior factor mediates the relationships among interpersonal factors and PA. Social network, social participation, family, neighborhood and school social capital, by strengthening autonomous PA motivation, delivered their positive effects for leisure time MVPA. For example, in line with the current study, in a study with middle school students, autonomous motivation (RAI) mediated the relationship of parental and school teachers’ involvement and students’ leisure PA [50]. It seems that motivation is an important factor of PA across ages in youth. So, paying attention to factors that strengthen PA motivation is crucial through lifetime and especially in youth. However, factors that are important for motivation may vary across ages. This is also concluded in a recent research review, which confirms that although adolescents’ autonomous motivation for PA is based on the social context, however during adolescence these social contexts can change frequently [51]. In contrast, some studies have not found the direct effect of RAI for PA, however RAI, representing quality of motivation was a significant moderator in the relationship of quantity of motivation and PA [52].

Among controlling socio-demographic factors, neither parental education, nor place of living or students’ age predicted meeting MVPA recommendations in the current study. Only male gender has emerged as an important predictor. The fact that adolescent boys are more physically active than girls is confirmed across many countries in the previous cross-national study [7].

Taken together, the current study assists for PA intervention planning within ecological framework. This study results are in line with the propositions of other authors that the most effective intervention strategy seems to lie at the personal level [53], and more specifically in strengthening PA motivation, which is amenable to change through social capital in the contexts important for adolescents—family, neighborhood, school—as well as encouraging development of social networks and social participation. The interaction effects of environmental factors with other PA determinants within an ecological framework should be further studied more specifically, incorporating indoor and outdoor recreation or exercise facilities, bike/hiking/walking trails, paths, basketball courts, running tracks, other playing fields and public parks [33]. 

### Strengths and Limitations

The advantage of the current study is that it represents a population of adolescents of a certain age with quite a large sample evaluated. The main limitation is that this was a cross-sectional study. The relationships identified here might have an inverse effect. For example, the reverse relationship between environmental factors and social capital could be expected [12]. People living in places that are friendly for PA (in terms of infrastructure and safety) might have greater opportunities for social interaction during their physical activity practices and thus build their social capital. These bidirectional effects are not considered here. There is also the issue of a bidirectional effect in PA motivation—MVPA’s relationship to past PA behavior might have an impact on further PA motivation [51]. Further, evaluation of environmental factors in the current study might have been too general and based on students’ perception rather than actual presence of PA facilities and infrastructure.

## 5. Conclusions

High school students’ leisure MVPA is directly related to environmental, interpersonal and personal factors: higher accessibility to physical activity recourses and higher neighborhood safety, higher family social capital, greater social network and social participation and higher physical activity motivation, respectively, while controlling for sociodemographic factors. Neither accessibility to physical activity recourses, nor neighborhood safety moderated the relationships among social capital, motivation and physical activity. However, physical activity motivation mediated the positive relationships among social capital components and physical activity. According to the results, future intervention strategies should focus on strengthening PA motivation by encouraging the development of social networks and social participation as well as family, neighborhood, and school social capital within the framework of an ecological model. That means the interventions should be holistic, including a community’s social (such as families of students, neighborhood) and physical environmental changes.

## Figures and Tables

**Table 1:** Descriptive statistics and results of the univariate and multivariate logistic regression models predicting meeting PA recommendation (≥7 h/week) of moderate-to-vigorous physical activity (MVPA) (*n* = 1285).

Study Variables	Descriptives	Univariate Logistic Regression		Multivariate Logistic Regression	
	Mean ± SD or %	OR [95% CI]	Nagelkerke R^2^	OR [95% CI]	Nagelkerke R^2^
**MVPA h/week**	3.81 ± 2.73	N/A	N/A	N/A	N/A
**MVPA (achieve ≥ 7 h/week)**	16.3	N/A	N/A	N/A	N/A
**Environmental**					
Neighborhood PA resources (high)	81.8	1.84 [1.13–2.99] *	0.03	1.74 [0.87–3.44]	0.16
Neighborhood safety (high)	76.5	1.54 [1.02–2.34] *		1.71 [0.98–2.98]	
**Interpersonal**					
Social network (range 0–25)	6.15 ± 5.15	1.05 [1.01–1.09] *	0.05	1.03 [0.99–1.07]	
Social participation (range 0–46)	9.01 ± 9.02	1.04 [1.02–1.06] **		1.03 [1.01–1.05] **	
Family SC (range 1–5)	4.07 ± (0.96	1.06 [1.01–1.11] *		1.04 [0.98–1.09]	
Neighborhood SC (range 1–5)	3.75 ± 1.08	1.07 [0.95–1.21]		0.98 [0.86–1.12]	
School/peers SC (range 1–5)	3.59 ± 0.93	0.97 [0.91–1.02]		0.96 [0.90–1.01]	
**Personal**					
RAI (range 15–20)	7.28 ± 6.74	1.12 [1.08–1.15] **	0.09	1.11 [1.06–1.15] **	
**Sociodemographic**					
Gender (male)	42.2	1.77 [1.28–2.44] **	0.004	1.71 [1.13–2.57] *	
Age (range 14–18)	16.14 ± 1.22	1.12 [0.97–1.27]		1.14 [0.96–1.33]	
Mother’ education (at least college)	59.6	1.05 [0.71–1.54]		0.92 [0.56–1.51]	
Fathers’ education (at least college)	46.9	1.19 [0.82–1.72]		1.20 [0.75–1.91]	
Place of living (rural)	38.9	1.00 [0.71–1.40]		1.08 [0.69–1.67]	

Note. *, *p* < 0.05; **, *p* < 0.01; N/A, not applicable; h, hours; PA, physical activity; SC, social capital; RAI, relative autonomy index.

**Table 2:** Gender-adjusted analysis of mediation effect of relative autonomy index for the relationship of social capital and MVPA.

Pathways from:	Effect	Pathway to RAI		Pathway to MVPA via RAI	
		β	95% CI	β	95% CI
Social Participation	Direct effect	2.02	[1.600–2.450] ***	0.04	[0.004–0.074] *
	Indirect effect			0.01	[0.005–0.022]
	CSIE			0.03	[0.010–0.041]
Social Network	Direct effect	1.30	[0.923–1.668] ***	0.05	[0.026–0.063] ***
	Indirect effect			0.01	[0.001–0.009]
	CSIE			0.02	[0.002–0.031]
Family Social Capital	Direct effect	1.48	[1.046–1.920] ***	0.10	[−0.097–0.293]
	Indirect effect			0.20	[0.138–0.282]
	CSIE			0.07	[0.048–0.096]
Neighborhood Social Capital	Direct effect	0.14	[0.610–0.221] ***	0.07	[−0.096–0.235]
	Indirect effect			0.13	[0.081–0.182]
	CSIE			0.05	[0.032–0.071]
School and Peers Social Capital	Direct effect	0.05	[0.006–0.094] *	0.09	[−0.105–0.281]
	Indirect effect			0.15	[0.090–0.214]
	CSIE			0.05	[0.030–0.071]

Note. Indicators in lines of Direct effect and Indirect effect contains unstandardized β coefficients; *, *p* < 0.05; ***, *p* < 0.001; MVPA, moderate to vigorous physical activity; RAI, relative autonomy index; CSIE, completely standardized indirect effect.

## Data Availability

The data presented in this study are available on request from the corresponding author.

## References

[1] McKinney J., Lithwick D.J., Morrison B.N., Nazzari H., Isserow S.H., Heilbron B., Krahn A.D. (2016). The health benefits of physical activity and cardiorespiratory fitness. BC Med. J..

[2] Poitras V.J., Gray C.E., Borghese M.M., Carson V., Chaput J.P., Janssen I., Katzmarzyk P.T., Pate R.R., Gorber S.C., Kho M.E. (2016). Systematic review of the relationships between objectively measured physical activity and health indicators in school-aged children and youth. Appl. Physiol. Nutr. Metab..

[3] Marques A., Demetriou Y., Tesler R., Gouveia É.R., Peralta M., Matos M.G. (2019). Healthy lifestyle in children and adolescents and its association with subjective health complaints: Findings from 37 countries and regions from the HBSC Study. Int. J. Environ. Res. Public Health.

[4] Farooq M.A., Parkinson K.N., Adamson A.J., Pearce M.S., Reilly J.K., Hughes A.R., Janssen X., Basterfield L., Reilly J.J. (2018). Timing of the decline in physical activity in childhood and adolescence: Gateshead Millennium Cohort Study. Br. J. Sports Med..

[5] Farooq A., Martin A., Janssen X., Wilson M.G., Gibson A.M., Hughes A., Reilly J.J. (2020). Longitudinal changes in moderate-to-vigorous-intensity physical activity in children and adolescents: A systematic review and meta-analysis. Obes. Rev..

[6] Corder K., Winpenny E., Love R., Brown H.E., White M., Van Sluijs E. (2019). Change in physical activity from adolescence to early adulthood: A systematic review and meta-analysis of longitudinal cohort studies. Br. J. Sports Med..

[7] Bann D., Scholes S., Fluharty M., Shure N. (2018). Physical activity in adolescence: Cross-national comparisons of levels, distributions and disparities across 52 countries. BioRxiv.

[8] Kalajas-Tilga H., Koka A., Hein V., Tilga H., Raudsepp L. (2019). Motivational processes in physical education and objectively measured physical activity among adolescents. J. Sport Health Sci..

[9] Dishman R.K., McIver K.L., Dowda M., Pate R.R. (2018). Declining physical activity and motivation from middle school to high school. Med. Sci. Sports Exerc..

[10] Chen W.L., Zhang C.G., Cui Z.Y., Wang J.Y., Zhao J., Wang J.W., Wang X., Yu J.M. (2019). The impact of social capital on physical activity and nutrition in China: The mediating effect of health literacy. BMC Public Health.

[11] Tabak I., Mazur J., Nałęcz H. (2017). Family and individual predictors and mediators of adolescent physical activity. Health Psychol. Rep..

[12] Mazumdar S., Learnihan V., Cochrane T., Davey R. (2018). The built environment and social capital: A systematic review. Environ. Behav..

[13] Salvy S.J., Feda D.M., Epstein L.H., Roemmich J.N. (2017). The social context moderates the relationship between neighborhood safety and adolescents’ activities. Prev. Med. Rep..

[14] Kärmeniemi M., Lankila T., Ikäheimo T., Koivumaa-Honkanen H., Korpelainen R. (2018). The built environment as a determinant of physical activity: A systematic review of longitudinal studies and natural experiments. Ann. Behav. Med..

[15] Sherif M., Sherif C.W. (1969). Social Psychology.

[16] Gubbels J.S., Van Kann D.H., de Vries N.K., Thijs C., Kremers S.P. (2014). The next step in health behavior research: The need for ecological moderation analyses-an application to diet and physical activity at childcare. Int. J. Behav. Nutr. Phys. Act..

[17] US Department of Health and Human Services (2005). Theory at a Glance: A Guide for Health Promotion Practice.

[18] Thornton C.M., Kerr J., Conway T.L., Saelens B.E., Sallis J.F., Ahn D.K., Frank L.D., Cain K.L., King A.C. (2017). Physical activity in older adults: An ecological approach. Ann. Behav. Med..

[19] Button B., Trites S., Janssen I. (2013). Relations between the school physical environment and school social capital with student physical activity levels. BMC Public Health.

[20] World Health Organization Global Strategy on Diet, Physical Activity and Health. https://www.who.int/dietphysicalactivity/physical_activity_intensity/en/.

[21] Motl R.W., Dishman R.K., Ward D.S., Saunders R.P., Dowda M., Felton G., Pate R.R. (2005). Perceived physical environment and physical activity across one year among adolescent girls: Self-efficacy as a possible mediator?. J. Adolesc. Health.

[22] Morrow V. (1999). Conceptualising social capital in relation to the well-being of children and young people: A critical review. Sociol. Rev..

[23] Parcel T.L., Dufur M.J., Cornell Zito R. (2010). Capital at home and at school: A review and synthesis. J. Marriage Fam..

[24] Furuta M., Ekuni D., Takao S., Suzuki E., Morita M., Kawachi I. (2012). Social capital and self-rated oral health among young people. Community Dent. Oral Epidemiol..

[25] Novak D., Kawachi I. (2015). Influence of different domains of social capital on psychological distress among Croatian high school students. Int. J. Ment. Health Syst..

[26] Stjernqvist N.W., Sabinsky M., Morgan A., Trolle E., Thyregod C., Maindal H.T., Bonde A.H., Tetens I. (2018). Building school-based social capital through ‘We Act-Together for Health’–a quasi-experimental study. BMC Public Health.

[27] Markland D., Tobin V. (2004). A modification to the behavioural regulation in exercise questionnaire to include an assessment of amotivation. J. Sport Exerc. Psychol..

[28] Markland D., Ingledew D.K. (2007). The relationships between body mass and body image and relative autonomy for exercise among adolescent males and females. Psychol. Sport Exerc..

[29] Muthén L.K., Muthén B.O. (1998–2017). Mplus User’s Guide.

[30] Hayes A.F. (2018). Partial, conditional, and moderated moderated mediation: Quantification, inference, and interpretation. Commun. Monogr..

[31] Preacher K.J., Kelley K. (2011). Effect size measures for mediation models: Quantitative strategies for communicating indirect effects. Psychol. Methods.

[32] Cohen J. (1992). A power primer. Psychol. Bull..

[33] D’Angelo H., Fowler S.L., Nebeling L.C., Oh A.Y. (2017). Adolescent physical activity: Moderation of individual factors by neighborhood environment. Am. J. Prev. Med..

[34] Ding D., Sallis J.F., Kerr J., Lee S., Rosenberg D.E. (2011). Neighborhood environment and physical activity among youth: A review. Am. J. Prev. Med..

[35] Weinberg D., Stevens G.W., Bucksch J., Inchley J., de Looze M. (2019). Do country-level environmental factors explain cross-national variation in adolescent physical activity? A multilevel study in 29 European countries. BMC Public Health.

[36] McNeill L.H., Kreuter M.W., Subramanian S.V. (2006). Social environment and physical activity: A review of concepts and evidence. Soc. Sci. Med..

[37] Golan M. (2006). Parents as agents of change in childhood obesity–from research to practice. Int. J. Pediatr. Obes..

[38] Pearson N., Timperio A., Salmon J., Crawford D., Biddle S.J. (2009). Family influences on children’s physical activity and fruit and vegetable consumption. Int. J. Behav. Nutr. Phys. Act..

[39] Kim H.H., Chun J. (2018). Analyzing multilevel factors underlying adolescent smoking behaviors: The roles of friendship network, family relations, and school environment. J. School Health.

[40] Novak D., Doubova S.V., Kawachi I. (2016). Social capital and physical activity among Croatian high school students. Public Health.

[41] Sevil J., García-González L., Abós Á., Generelo Lanaspa E., Aibar Solana A. (2018). Which school community agents influence adolescents’ motivational outcomes and physical activity? Are more autonomy-supportive relationships necessarily better?. Int. J. Environ. Res. Public Health.

[42] Macdonald-Wallis K., Jago R., Sterne J.A. (2012). Social network analysis of childhood and youth physical activity: A systematic review. Am. J. Prev. Med..

[43] Pedditzi M.L., Marcello P. (2018). School social context, students’ self-efficacy and satisfaction in high school. Open Psychol. J..

[44] Yıldızer G., Bilgin E., Korur E.N., Novak D., Demirhan G. (2018). The association of various social capital indicators and physical activity participation among Turkish adolescents. J. Sport Health Sci..

[45] Rhodes R.E., Saelens B.E., Sauvage-Mar C. (2018). Understanding physical activity through interactions between the built environment and social cognition: A systematic review. Sports Med..

[46] Brand R., Cheval B. (2019). Theories to explain exercise motivation and physical inactivity: Ways of expanding our current theoretical perspective. Front. Psychol..

[47] Barbeau A., Sweet S.N., Fortier M. (2009). A path-analytic model of self-determination theory in a physical activity context. J. Appl. Biobehav. Res..

[48] Gunnell K.E., Crocker P.R., Mack D.E., Wilson P.M., Zumbo B.D. (2014). Goal contents, motivation, psychological need satisfaction, well-being and physical activity: A test of self-determination theory over 6 months. Psychol. Sport Exerc..

[49] Mahony R., Blake C., Matthews J., O’Donnoghue G., Cunningham C. (2018). Physical activity levels and self-determined motivation among future healthcare professionals: Utility of the Behavioral Regulation in Exercise Questionnaire (BREQ-2). Physiother. Theory Pract..

[50] McDavid L., Cox A.E., Amorose A.J. (2012). The relative roles of physical education teachers and parents in adolescents’ leisure-time physical activity motivation and behavior. Psychol. Sport Exerc..

[51] Palmer K., Robbins L.B., Ling J., Kao T.S., Voskuil V.R., Smith A.L. (2020). Adolescent autonomous motivation for physical activity: A concept analysis. J. Pediatr. Nurs..

[52] Fortier M.S., Wiseman E., Sweet S.N., O’Sullivan T.L., Blanchard C.M., Sigal R.J., Hogg W. (2011). A moderated mediation of motivation on physical activity in the context of the Physical Activity Counseling randomized control trial. Psychol. Sport Exerc..

[53] Mehtälä M.A., Sääkslahti A.K., Inkinen M.E., Poskiparta M.E. (2014). A socio-ecological approach to physical activity interventions in childcare: A systematic review. Int. J. Behav. Nutr. Phys. Act..

